# Prevalence, Awareness, and Control of Arterial Hypertension in a Russian Population. The Ural Eye and Medical Study

**DOI:** 10.3389/fpubh.2019.00394

**Published:** 2020-01-08

**Authors:** Mukharram M. Bikbov, Gyulli M. Kazakbaeva, Rinat M. Zainullin, Venera F. Salavatova, Timur R. Gilmanshin, Dilya F. Yakupova, Yulia V. Uzianbaeva, Inga I. Arslangareeva, Songhomitra Panda-Jonas, Svetlana R. Mukhamadieva, Renat I. Khikmatullin, Said K. Aminev, Ildar F. Nuriev, Artur F. Zaynetdinov, Jost B. Jonas

**Affiliations:** ^1^Ufa Eye Research Institute, Ufa, Russia; ^2^Department of Ophthalmology, Medical Faculty Mannheim of the Ruprecht-Karls-University of Heidelberg, Mannheim, Germany

**Keywords:** arterial hypertension, elevated blood pressure, hypertension, blood pressure, epidemiology, population-based study, Russia, Ural Eye and Medical Study

## Abstract

**Background:** Applying the criteria recently published by the American College of Cardiology/American Heart Association for the definition of arterial hypertension, we investigated prevalence and awareness of arterial hypertension in Russia. This new definition differentiates between normal BP [SBP (systolic blood pressure)/DBP (diastolic blood pressure) < 120/80 mmHg], elevated BP (SBP 120–129 mmHg; DBP < 80 mmHg), hypertension stage 1 (SBP 130–139 mmHg or DBP 80–89 mmHg), hypertension stage 2 (SBP ≥ 140 and ≤180 mmHg or DBP ≥ 90 and ≤120 mm Hg) and hypertensive crisis (SBP > 180 mmHg and/or DBP > 120).

**Methods:** The population-based Ural Eye and Medical Study, performed in an urban and rural region in the Russian republic Bashkortostan, included 5,891 (80.5%) individuals aged 40+ years out of 7,328 eligible individuals. The participants underwent a detailed interview and medical examination. Arterial hypertension was defined using the criteria defined by the American College of Cardiology/American Heart Association.

**Results:** The prevalence of normal blood pressure (BP), elevated BP, hypertension stage 1, stage 2, and hypertensive crisis was 750/5,891 [12.7%; 95% confidence interval (CI): 4.7, 5.9], 312/5, 891 (5.3%; 95% CI: 4.7, 5.9), 2,187/5,891 (37.1%; 95% CI: 35.9, 38.4), 2,484/5,891 (42.2%; 95% CI: 40.9, 43.4), and 158/5,891 (2.7%; 95% CI: 2.3, 3.1), respectively. The overall prevalence of elevated BP/hypertension was 5,141/5,891 (87.3%; 95% CI: 86.4, 88.1). Awareness of elevated BP/hypertension was 2,289/5,223 (45.4%; 95% CI: 44.0, 47.0). Among 1,055 (20.2%; 95% CI: 19.1, 21.3) individuals under anti-hypertensive treatment, 33 (3.1%) individuals had normal BP values. Higher risk of elevated BP/hypertension was associated with older age [odds ratio (OR): 1.04; 95% CI: 1.03,1.05], male gender (OR: 2.56; 95% CI: 2.10, 3.16), urban region (OR: 1.26; 95% CI: 1.05, 1.51), lower educational level (OR: 0.92; 95% CI: 0.87, 0.97), higher body mass index (OR: 1.15; 95% CI: 1.12, 1.18), higher waist-hip circumference ratio (OR: 6.16; 95% CI: 1.89, 20.0), higher prevalence of sitting or reclining for more than 18 h per week (OR: 1.33; 95% CI: 1.10, 1.61), higher prevalence of alcohol consumption (OR: 1.61; 95% CI: 1.27, 2.05), and higher serum concentrations of triglycerides (OR: 1.22; 95% CI: 1.05, 1.43) and glucose (OR: 1.15; 95% CI: 1.07, 1.24). Using the former definition of hypertension (systolic BP ≥ 140 mmHg and/or diastolic BP ≥ 90 mmHg), the prevalence of hypertension was 3,134/5,891 (53.2%; 95% CI: 51.9, 54.5).

**Conclusions:** Using the new definition of arterial hypertension, the prevalence of elevated BP/hypertension in a typically mixed Russian population aged 40+ years was high (87.3%), with an awareness rate of 45.4% and treatment rate of 20.2%. The rate of therapeutic control of BP elevation in the individuals under treatment was <5%.

## Introduction

Increased systolic blood pressure (SBP) is one of the three leading risk factors for disability-adjusted life-years (DALYs), as examined in the Global Burden of Diseases, Injuries, and Risk Factors (GBD) 2016 Study (1). It caused 122.2 million [95% uncertainty interval (UI): 110.3–133.3 million] DALYs for men and 89.9 million DALYs (95% UI: 80.9–98.2 million) for women. Another study by the GBD revealed that the prevalence of SBP of at least 110–115 mm Hg increased in the period from 1990 to 2015 from 73,119/100,000 to 81,373/100,000, and that the prevalence of SBP of 140 mm Hg or higher increased from 17,307/100,000 to 20,526/100,000 (2). The annual death rate per 100,000 related to a SBP of at least 110–115 mm Hg increased from 135.6 to 145.2, and the annual death rate per 100,000 related to a SBP of 140 mm Hg or higher increased from 97.9 to 106.3.

Despite the general importance of elevated blood pressure and arterial hypertension for public health, information about the occurrence of hypertension in Russia and factors associated with the prevalence of hypertension in Russia have been scarce so far (3–6). This may hold true even more if one considers that Russia is the largest country worldwide with one of the largest populations. Although a recent meta-analysis estimated that for both sexes of all ages, high SBP was the leading mortality risk factor causing 32.7% of all deaths in Russia in 2016, the basis for these estimations has remained small so far (3–6). Two studies performed by Mozheyko et al. in 2011 and 2012 examined patients with hypertension and attending government-run outpatient health facilities (5, 6), while a primary recent population-based study on the prevalence of arterial hypertension in Russia has not been available. That was the reason to perform the present investigation with the aim of exploring the prevalence of arterial hypertension in a population in Russia and to assess the associations of arterial hypertension with other parameters.

## Methods

The Ural Eye and Medical Study is a cross-sectional population-based cohort study that was conducted in the city of Ufa as the capital of the republic of Bashkortostan and in villages in a distance of 65 km from Ufa in the period from October 2015 till July 2017 (7–9). The Ethics Committee of the Academic Council of the Ufa Eye Research Institute declared that the study was conducted in agreement with the Declaration of Helsinki and approved the study protocol. An informed written consent was given by all participants. The population of Ufa includes Russians, Tatars, Bashkirs, Ukrainians, and other ethnicities. Bashkortostan is situated at southern Ural Mountains about 1,300 km East of Moscow. Living in the study region and an age of 40+ years were the inclusion criteria, while the study did not have any exclusion criterion. The study included 5,899 (80.5%) out of 7,328 eligible individuals (mean age: 59.0 ± 10.7 years; range: 40–94 years).

The study participants underwent a standardized interview with more than 250 questions on socioeconomic parameters, diet, tobacco consumption, daily physical activity, alcohol consumption, depression and suicidal ideas, and medical history. The interview was conducted by trained social workers. We applied the “guidelines for accurate and transparent health estimates reporting” for the collection and reporting of the data (10). The series of examinations started with the measurement of arterial blood pressure (BP), pulse and anthropometric parameters. The BP was determined with the individual sitting for at least 5 min. The individuals had not smoked or taken any coffee, tea, or alcohol for at least 3 h, nor had they undertaken any physical exercise for half an hour before the BP measurements were taken. We measured the blood pressure three times, and we took the mean value for further statistical analysis. We used an automatic tonometer (OMRON M2, Omron Co., Kyoto, Japan) the cuff size of which was adapted to the upper arm circumference.

Applying the new guidelines of the American College of Cardiology and the American Heart Association for the detection, prevention, management, and treatment of high BP, we differentiated between normal BP [SBP/DBP (diastolic blood pressure) <120/80 mm Hg], elevated BP (SBP between 120 and 129 mm Hg and DBP <80 mm Hg), stage 1 of hypertension (SBP between 130 and 139 mm Hg or DBP between 80 and 89 mm Hg), stage 2 of hypertension (SBP ≥140 and ≤180 mm Hg or DBP ≥90 and ≤120 mm Hg) and a hypertensive crisis (SBP > 180 mm Hg and/or DBP > 120) (11, 12). This new definition no longer contains the category of arterial prehypertension which now has been split up into “elevated BP” or “stage I of hypertension.” Awareness of elevated BP/hypertension was defined as the knowledge about the disorder affecting the individual study participant.

Additional procedure performed included dynamometric handgrip strength measurement, biochemical analysis, and fasting blood samples and spirometry. We defined diabetes mellitus by a glucose concentration of ≥7.0 mmol/L or a self-reported history of a physician-based diagnosis of diabetes mellitus or a history of drug treatment for diabetes. We used the Center for Epidemiologic Studies Depression Scale (CES-D) scoresheet for the assessment of depression, and the State-Trait Anxiety Inventory (STAI) to explore trait and state anxiety. The study has been described in detail recently (7–9).

A statistical software program (Statistical Package for Social Science, SPSS, version 25.0; IBM-SPSS Inc., Chicago, USA) was applied for the statistical analysis. We calculated the mean BP values expressed as mean ± standard deviation, and the mean prevalence of hypertension and its various stages, presented as mean and 95% confidence intervals (CI). We looked for associations between the prevalence of hypertension and other parameters, firstly in univariate analyses, then in a multivariable binary regression analysis. The multivariable analysis included as dependent variable the presence of elevated BP/hypertension and as independent variables all those parameters that were significantly associated with the prevalence of elevated BP/hypertension in the univariate analysis. We then dropped, step by step, all those parameters which were no longer significantly associated with the dependent variable. We used a multivariable linear regression analysis to explore relationships between the BP readings and other parameters. This multivariable linear regression analysis included the BP reading as dependent variable and as independent variables those parameters which were significantly correlated with the BP reading in the univariate analysis. We then dropped all those parameters either showing a high collinearity or which were no longer significantly associated with the physical activity score. We determined odds ratios (OR) and their 95% confidence intervals (CI). We considered two-sided *P*-values as statistically significant if they were smaller than 0.05.

## Results

Out of the 5,899 participants of the Ural Eye and Medical Study, 5,891 (99.9%) individuals had BP measurements. With 99.9% of the original study population participating in the present investigation, the group of individuals with information on BP as compared with the group of subjects without examination of the BP did not differ significantly in age (59.0 ± 10.7 vs. 66.5 ± 13.7 years; *P* = 0.16) and level of education (*P* = 0.47). The group of non-participants had a significantly higher percentage of women [2,580 (43.8%) men/3,311 (56.2%) women vs. 0 (0%) men/8 (100%) women; *P* = 0.01]. The composition of the study population with respect to gender and age corresponded to the gender and age distribution in the Russian population according to the most recent census carried out in 2010 (https://www.gks.ru/). Mean body mass index was 27.9 ± 5.0 kg/m^2^ (median: 27.3 kg/m^2^; range: 13.96–60.96 kg/m^2^) and the mean waist-hip ratio was 0.91 ± 0.09 (median: 0.91; range: 0.44–1.84).

Mean SBP was 133.6 ± 20.5 mmHg (range: 83–232 mmHg), and mean DBP was 82.0 ± 10.4 mm Hg (range: 40–154 mm Hg). Mean BP was 99.2 ± 12.5 mm Hg (median: 96.7 mmHg; range: 54.7–165.3 mmHg). For 3,204 randomly selected participants, the BP as measured at both upper arms, did not show a significant difference between the right arm and the left arm for the systolic measurements (133.5 ± 20.0 vs. 133.3 ± 20.1 mm Hg; *P* = 0.17), while the DBP was higher measured at the right upper arm than at the left upper arm (82.6 ± 10.3 vs. 82.2 ± 11.00 mm Hg: *P* < 0.001).

**Figure 1:**
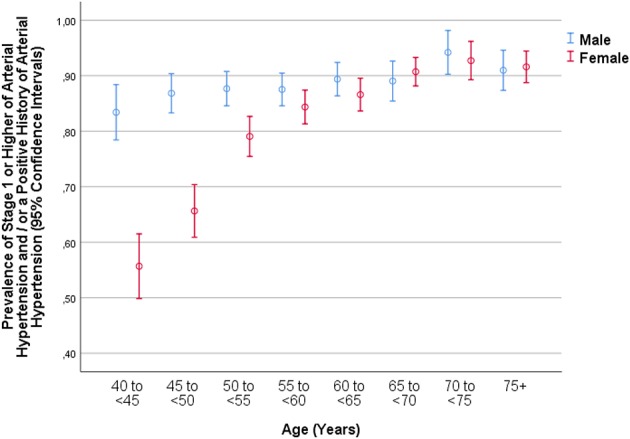
Graph showing the distribution of stage 1 or higher of arterial hypertension in the Ural Eye and Medical Study, stratified by age, and gender.

Normal BP (SBP/DBP < 120/80 mm Hg) was present in 750 (12.7%) of the study participants, elevated BP (SBP between 120 and 129 mm Hg and DBP < 80 mm Hg) in 312 (5.3%; 95% CI: 4.7, 5.9) individuals, stage 1 of hypertension (SBP between 130 and 139 mm Hg or DBP between 80 and 89 mm Hg) in 2,187 (37.1%; 95% CI: 35.9, 38.4) participants, stage 2 of hypertension (SBP ≥ 140 and ≤180 mm Hg or DBP ≥ 90 and ≤ 120 mm Hg) in 2,484 (42.2%; 95% CI: 40.9, 43.4) individuals, and a hypertensive crisis (SBP > 180 mm Hg and/or DBP >120) in 158 (2.7%; 95% CI: 2.3, 3.1) participants (Table 1, Figure 1). Prevalence of any elevated BP including the various stages of hypertension was 5,141/5,891 or 87.3% (95% CI: 86.4, 88.1). The prevalence of hypertension using its former definition as a SBP of ≥140 mm Hg and/or DBP of ≥90 mm Hg was present in 3,134/5,891 or 53.2% (95% CI: 51.9, 54.5). Including only study participants with Russian ethnicity revealed similar figures as for the total study population (Table 1). The prevalence of any elevated BP including the various stages of hypertension (new definition) was 87.3% (95% CI: 86.4, 88.1).

**Table 1:** Prevalence of elevated blood pressure or stage 1 or higher of arterial hypertension in the Ural Eye and Medical Study, stratified by age and gender.

	**Men**		**Women**		*P*-value^*^	**Total study population**		**Russian subgroup**	
**Age (Years)**	***n***	**Prevalence [%; 95% Confidence Interval (CI)]**	***n***	**Prevalence (%; 95% CI)**		***n***	**Prevalence (%; 95% CI)**	***n***	**Prevalence (%; 95% CI)**
40 to <45	195/217	89.9 (85.8, 93.9)	181/282	64.2 (58.6, 69.8)	<0.001	376/499	75.4 (71.6, 79.2)	80/106	75.5 (67.2, 83.8)
45 to <50	327/357	91.6 (88.7, 94.5)	270/387	69.8 (65.2, 74.4)	<0.001	597/744	80.2 (77.4, 83.1)	116/147	78.9 (72.2, 85.6)
50 to <55	402/438	91.8 (89.2, 94.4)	400/492	81.3 (77.8, 84.8)	<0.001	802/930	86.2 (84.0, 88.5)	126/141	89.4 (84.2, 94.5)
55 to <60	444/489	90.8 (88.2, 93.4)	470/550	85.5 (82.5, 88.4)	0.01	914/1,039	88.0 (86.0, 90.0)	139/162	85.8 (80.4, 91.2)
60 to <65	378/405	93.3 (90.9, 95.8)	448/515	87.0 (84.1, 89.9)	0.002	826/920	89.8 (87.8, 91.7)	178/194	91.8 (87.8, 95.7)
65 to <70	271/292	92.8 (89.8, 95.8)	450/496	90.7 (88.2, 93.3)	0.36	721/788	91.5 (89.6, 93.5)	192/208	92.3 (88.7, 96.0)
70 to <75	132/138	95.7 (92.2, 99.1)	207/220	94.1 (91.0, 97.2)	0.63	339/358	94.7 (92.4, 97.0)	86/93	92.5 (87.0, 97.9)
75+	229/244	93.9 (90.8, 96.9)	337/369	91.3 (88.4, 94.2)	0.28	566/613	92.3 (90.2, 94.4)	124/134	92,5 (88.0, 97.0)
Total	2,378/2,580	92.2 (91.1, 93.2)	2,763/3,311	83.4 (82.2, 84.7)	<0.001	5,141/5,891	87.3 (86.4, 88.1)	1,041/1,185	87.9 (86.0, 89.7)

* *P-value: statistical significance of the difference between men and women*.

Hypertension was known for 2,371 (40.3%; 95% CI: 39.0, 42.0) individuals of the study participants. Defining hypertension as stage 1 or higher of hypertension and/or a history of and/or therapy of hypertension, its prevalence was 4,979/5,891 or 84.5% (95% CI: 83.6, 85.4). Among these participants with stage 1 or higher of hypertension or history of it (*n* = 4,979), 2,371 (47.6%; 95% CI: 46.0, 49.0) were aware of having elevated BP, and 1,055 (21.2%; 95% CI: 20.1, 22.3) were on anti-hypertensive treatment. Among the participants with any elevated BP, stage 1 or higher of hypertension or known hypertension (*n* = 5,223), 2,289 (45.4%; 95% CI: 44.0, 47.0) were aware of having elevated BP, and 1,055 (20.2%; 95% CI: 19.1, 21.3) were on anti-hypertensive treatment.

In the whole study population, anti-hypertensive therapy by oral medication was taken by 1,055 individuals or 17.9% (95% CI: 16.9, 18.9) of the whole study population or 44.5% (95% CI: 42.5, 46.5) of the patients with known hypertension. Among those with anti-hypertensive therapy, 33 (3.1%) individuals had normal BP values, 20 (1.9%) individuals had elevated BP, 230 (21.8%) participants had stage 1 of hypertension, 713 (67.6%) individuals had stage 2 of hypertension, and 59 (5.6%) participants had a hypertensive crisis.

In a multivariable binary logistic regression analysis, a higher prevalence of elevated BP/hypertension (i.e., any SBP ≥ 120 mm Hg or any DBP ≥ 80 mm Hg as the cut-off values) was associated with older age (OR: 1.04; *P* < 0.001), male gender (OR: 2.56; *P* < 0.001), urban region of habitation (OR: 1.26; *P* = 0.01), higher blood concentrations of triglycerides (OR: 1.22; *P* = 0.01), and glucose (OR: 1.15; *P* < 0.001), lower level of education (OR: 0.92; *P* = 0.003), higher prevalence of alcohol consumption (OR: 1.61; *P* < 0.001), higher body mass index (OR: 1.15; *P* < 0.001), higher waist-hip circumference ratio (OR: 6.16; *P* = 0.003), and sitting or reclining for more than 18 h per week (OR: 1.33; *P* = 0.003) (Tables 2, 3). In that model, other parameters were not significantly correlated with the prevalence of elevated BP/hypertension; it included the variables of current smoking (*P* = 0.86) and smoking package years (*P* = 0.97), anxiety score (*P* = 0.64), depression score (*P* = 0.73), the spirometric forced vital capacity in 1 s (*P* = 0.48) and forced expiratory volume (*P* = 0.88), dietary parameters such as number of days with fruit intake (*P* = 0.08) or vegetable intake (*P* = 0.57), ethnicity (Russian vs. non-Russian) (*P* = 0.73), family status (married vs. non-married) (*P* = 0.11), hearing loss score (*P* = 0.73), religion (Muslim vs. non-Muslim) (*P* = 0.90), and biochemical blood parameters such as blood concentration of hemoglobin (*P* = 0.30), high-density lipoproteins (*P* = 0.74), and low-density lipoproteins (*P* = 0.16), and the erythrocyte cell count (*P* = 0.31) (Figure 1).

**Table 2:** Demographic, socioeconomic, lifestyle-associated, and other parameters (mean ± standard deviations; frequency and 95% confidence intervals) in the Ural Eye ad Medical Study stratified by the presence of elevated blood pressure/arterial hypertension.

**Parameter**	**Elevated blood pressure/arterial hypertension**	**Normal blood pressure**	***P*-value**
*n*	5,141	750	
Age (years)	59.6 ± 10.6	54.9 ± 10.6	<0.001
Men/women	2,378 (46.3%)/2,763 (53.7%)	202 (26.9%)/548 (73.1%)	<0.001
Urban/rural region of habitation	2,970 (57.8%)/2,171 (42.2%)	429 (57.2%)/321 (42.8%)	0.78
Family status: married/unmarried/divorced/widowed/missing	3,757 (73.1%)/321 (6.2%)/282 (5.5%)/778 (15.1%)	553 (73.7%)/55 (7.3%)/55 (7.3%)/87 (11.6%)	0.18
Family status: married vs. any other status	3,757 (73.1%)/1,381 (26.9%)	553 (73.7%)/197 (26.3%)	0.76
Family type: joint (three generations)/nuclear (two generations)/single/family of two people	1,355 (26.4%)/2,163 (42.2%)/284 (5.5%)/1,321 (25.8%)	186 (24.9%)/337 (45.1%)/40 (5.3%)/185 (24.7%)	0.87
Religion: Muslim/Christian/other	3,197 (62.2%)/1852 (36.0%)/91 (1.8%)	470 (62.7%)/267 (35.6%)/13 (1.7%)	0.81
Religion: Muslim/any other religion	3,197 (62.2%)/1,943 (37.8%)	470 (62.7%)/280 (37.3%)	0.84
Ethnicity: Russian/Bashkirs/Tatars/Chuvash/Mari/others/missing	1,041 (22.1%)/921 (19.5)/2,141 (45.4%)/510 (10.8%)/18 (0.4%)/82 (1.7%)	144 (21.1%)/139 (20.4%)/298 (43.6%)/77 (11.3%)/3 (0.4%)/22 (3.2%)	0.31
Ethnicity: Russian/any other ethnicity	1,041 (22.1%)/3,672 (71.4%)	144 (21.1%)/539 (78.9%)	0.59
Body height (cm)	165.0 ± 8.9	163.5 ± 8.0	<0.001
Body weight (kg)	77.1 ± 14.5	67.7 ± 11.9	<0.001
Body mass index (kg/m^2^)	28.3 ± 5.0	25.3 ± 4.2	<0.001
Waist circumference (cm)	95.1 ± 13.2	86.3 ± 12.0	<0.001
Hip circumference (cm)	104.1 ± 12.6	99.7 ± 11.5	<0.001
Waist/hip circumference ratio	0.92 ± 0.09	0.87 ± 0.09	<0.001
**SOCIOECONOMIC PARAMETERS**			
Level of education	5.5 ± 1.4	5.8 ± 1.8	<0.001
Monthly Income (Below poverty line/average/above average/high)	1,129 (22.0%)/3773 (73.4%)/228 (4.4%)/7 (0.1%)	188 (25.1%)/532 (70.9%)/30 (4.0%)/0 (0%)	0.048
Socioeconomic score	5.8 ± 1.5	6.1 ± 1.3	<0.001
Physical activity score	7.9 ± 8.1	8.0 ± 8.0	0.73
Over the past 7 days, how much time did you spend sitting or reclining on a typical day?	1,086 ± 940	937 ± 769	<0.001
**HISTORY OF DISEASES**			
History of arthritis	1,431 (27.8%)/3,709 (72.2%)	202 (26.9%)/548 (73.1%)	0.63
History of headache	2,203 (46.7%)/2,510 (53.3%)	344 (50.4%)/339 (49.6%)	0.08
History of neck pain	1,323 (28.1%)/3,390 (71.9%)	247 (36.2%)/436 (63.8%)	<0.001
History of thoracic spine pain	1,063 (22.6%)/3,650 (77.4%)	207 (30.3%)/476 (63.8%)	<0.001
History of back pain	2,513 (53.3%)/2,200 (46.7%)	398 (58.3%)/285 (41.7%)	0.02
History of therapy of hyperlipidemia	388 (8.7%)/4,050 (91.3%)	48 (7.7%)/575 (92.3%)	0.47
History of cancer	155 (3.0%)/4,985 (97.0%)	18 (2.4%)/732 (97.6%)	0.42
History of cardiovascular disorders including stroke	1,318 (28.0%)/3,395 (72.0%)	150 (22.0%)/533 (78.0%)	0.001
History of dementia	33 (0.7%)/4,680 (99.3%)	4 (0.6%)/679 (99.4%)	1.00
History of diabetes mellitus	468 (9.1%)/4,672 (90.9%)	31 (4.1%)/719 (95.9%)	<0.001
History of diarrhea	21 (0.4%)/4,692 (99.6%)	6 (0.9%)/677 (99.1%)	0.14
History of bone fracture	1,466 (31.1%)/3,247 (68.9%)	184 (26.9%)/499 (73.1%)	0.03
History of heart attack	287 (5.6%)/4,853 (94.4%)	27 (3.6%)/723 (96.4%)	0.02
History of iron-deficiency anemia	228 (4.8%)/4,485 (94.4%)	76 (11.1%)/607 (88.9%)	<0.001
History of low blood pressure and hospital admittance	188 (3.7%)/4,927 (96.3%)	27 (3.6%)/721 (96.4%)	1.00
History of osteoarthritis	845 (17.9%)/3,868 (82.1%)	144 (21.1%)/539 (78.9%)	0.05
History of skin disease	234 (5.0%)/4,479 (95.0%)	53 (7.8%)/630 (92.2%)	0.003
History of use of steroids	29 (0.6%)/5,107 (99.4%)	5 (0.7%)/749 (99.3%)	0.61
History of thyreopathy	513 (10.0%)/4,627 (90.0%)	96 (12.8%)/654 (87.2%)	0.02
History of tumbling	970 (18.9%)/4,166 (81.1%)	131 (17.5%)/619 (82.5%)	0.37
History of unconsciousness	410 (8.0%)/4,730 (92.0%)	79 (10.5%)/671 (89.5%)	0.02
Age of the last menstrual bleeding (years)	48.4 ± 5.0	47.9 ± 5.1	0.12
Age of last regular menstrual bleeding (years)	48.2 ± 5.0	47.7 ± 5.1	0.13
Menopause	2,044 (84.0%)/390 (16.0%)	307 (64.0%)/173 (36.0%)	<0.001
Blood concentrations (mmol/L) of:			
Alanine aminotransferase (IU/L)	21.4 ± 12.4	19.6 ± 9	<0.001
Aspartate aminotransferase (IU/L)	21.0 ± 11.4	19.4 ± 8.1	<0.001
Bilirubin, total (μmol/L)	15.0 ± 11.3	14.5 ± 10.8	0.32
High-density lipoproteins (mmol/L)	2.30 ± 0.90	2.41 ± 0.87	0.004
Low-density lipoproteins (mmol/L)	2.14 ± 1.21	2.06 ± 1.13	0.12
Triglycerides (mmol/L)	1.44 ± 0.77	1.23 ± 0.53	<0.001
Cholesterol (mmol/L)	5.82 ± 1.71	5.62 ± 1.49	0.003
Erythrocyte sedimentation rate (mm/h)	14.1 ± 11.3	14.9 ± 10.9	0.059
Glucose (mmol/L)	5.09 ± 1.73	4.64 ± 1.13	<0.001
Creatinine (μmol/L)	90.1 ± 23.0	88.5 ± 35.2	0.09
Urea (mmol/L)	5.12 ± 1.46	5.00 ± 1.46	0.03
Residual nitrogen (g/L)	0.25 ± 0.08	0.25 ± 0.05	0.18
Total protein (g/L)	76.0 ± 6.36	76.0 ± 6.14	0.85
International normalized ratio (INR)	1.06 ±0.14	1.07 ± 0.18	0.09
Blood clotting time (min)	3.74 ± 0.53	3.85 ± 0.55	<0.001
Prothrombin index (%)	96.1 ± 10.2	95.3 ± 10.5	0.057
Hemoglobin (g/dL)	143.2 ± 14.7	138.9 ± 15.0	<0.001
Erythrocytes (10^6^ cells/μL)	4.50 ± 0.38	4.40 ± 0.40	<0.001
Leukocytes (10^9^ cells/L)	5.14 ± 1.43	5.00 ± 1.44	0.02
Ankle-brachial index, right side	1.25 ± 0.17	1.41 ± 0.25	<0.001
Ankle-brachial index, left side	1.25 ± 0.18	1.37 ±0.24	<0.001
Vegetarian diet/mixed diet	7 (0.1%)/5,134 (99.9%)	3 (0.4%)/747 (99.6%)	0.12
Number of meals per day	3.6 ± 0.8	3.7 ± 0.8	0.005
In a week how many days do you eat fruits?	5.3 ± 2.0	5.5 ± 2.0	0.007
How many servings of fruit do you take on one of those days (g)	171 ± 108	167 ± 100	0.39
In a week how many days do you eat vegetables?	6.3 ± 1.4	6.3 ± 1.4	0.23
How many servings of vegetables do you eat on one of those days (gram)?	225 ± 128	219 ± 118	0.23
Type of oil used for cooking: vegetable oil/non-vegetable oil	3,897 (96.7%)/132 (3.2%)	566 (96.8%)/19 (3.2%)	1.00
Food containing whole grains (Yes/No)	3,726 (79.1%)/984 (20.9%)	565 (82.7%)/118 (17.3)	0.03
Salt consumed per day (g)	4.3 ± 2.4	4.3 ± 2.4	0.95
Degree of processing of meat (weak/medium/well done)	101 (2.1%)/1,681 (35.7%)/2,927 (62.2%)	18 (2.6%)/237 (34.8%)/427 (62.6%)	0.99
Do you currently smoke any tobacco products? (yes)	660 (12.9%)/4,474 (87.1%)	85 (11.3%)/665 (88.7%)	0.26
Do you smoke daily? (yes/no)	640 (12.4%)/4,501 (87.6%)	78 (10.4%)/672 (89.6%)	0.12
Package years (package = 20 cigarettes)	4.2 ± 13.0	3.2 ± 11.0	0.04
Alcohol consumed such as beer, whisky, rum, gin brandy or other local products? (yes/no)	1,151 (22.4%)/3,987 (77.6%)	106 (14.1%)/644 (85.9%)	<0.001
Hearing loss total score	5.3 ± 11.2	3.9 ± 9.3	0.002
Depression score (adapted)	1.16 ± 3.71	1.34 ± 3.90	0.22
State-Trait Anxiety Inventory (STAI) score	−0.67 ± 3.54	−0.58 ± 3.58	0.49
Manual dynamometry, right hand (dekaNewton)	30.8 ± 11.9	28.8 ± 10.1	<0.001
Manual dynamometry, left hand (dekaNewton)	27.2 ± 11.5	25.4 ± 9.8	<0.001

**Table 3:** Multivariable analysis of the associations between the prevalence of elevated blood pressure/arterial hypertension as outcome parameter and other systemic parameters as dependent parameters in the Ural Eye and Medical Study^*^.

**Parameter**	**Odds ratio**	**95% confidence interval**
Age: Per unit (years) increase in age	1.04	1.03, 1.05
Gender (men vs. women)	2.56	2.10, 3.16
Region of habitation (urban vs. rural)	1.26	1.05, 1.51
Blood concentration of triglycerides: Per unit (mmol/L) increase in triglycerides concentration	1.22	1.05, 1.43
Blood concentration of glucose: Per unit (mmol/L) increase in glucose concentration	1.15	1.07, 1.24
Level of education: Per unit (levels from 0 to 6) increase in educational level^**^	0.92	0.87, 0.97
Alcohol consumption (any alcohol consumption vs. no consumption)	1.61	1.27, 2.05
Body mass index: Per unit (kg/m^2^) increase in body mass index	1.15	1.12, 1.18
Waist-hip circumference ratio: Per unit increase in waist-hip ratio	6.16	1.89, 20.0
Sitting or reclining for more than 18 h per week (sitting for >18 h per week vs. sitting for ≤18 h)	1.33	1.10, 1.61

* *Elevated blood pressure was defined as systolic blood pressure (SBP) between 120 and 129 mm Hg and diastolic blood pressure (DBP) <80 mm Hg, and hypertension was defined as SBP ≥130 mm Hg and/or DBP ≥80 mm Hg*.

** *The level of education was categorized into the stages of “illiteracy” (no reading ability at all), “passing of the 5th class,” “passing of the 8th class,” “passing of the 10th class,” “passing of the 11th class,” “graduation,” and “post-graduation”*.

*The multivariable analysis included as dependent variable the presence of elevated BP/hypertension and as independent variables all those parameters that were significantly associated with the prevalence of elevated BP/hypertension in the univariate analysis. We then dropped, step by step, all those parameters which were no longer significantly associated with the dependent variable*.

**Figure 2:**
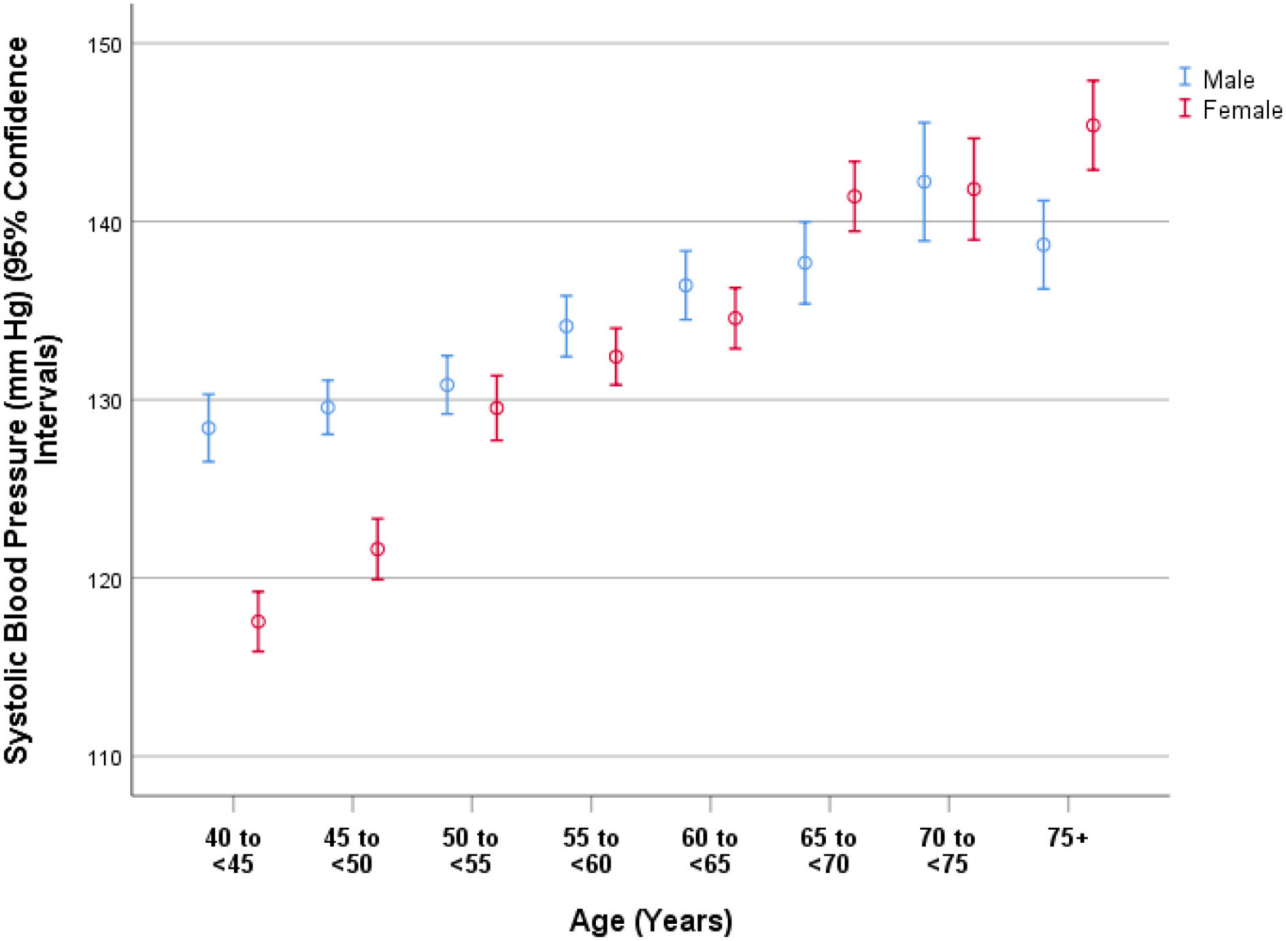
Graph showing the distribution of systolic blood pressure in the Ural Eye and Medical Study, stratified by age and gender.

In a similar manner, higher SBP was correlated (multivariable linear regression analysis) with older age (beta: 0.27) (Figure 2), male gender (beta: 0.06), lower level of education (beta: −0.03), higher blood concentrations of triglycerides (beta: 0.04) and glucose (beta: 0.07), higher prevalence of alcohol consumption (beta: 0.03), higher body mass index (beta: 0.24), higher waist-hip circumference ratio (beta: 0.07), and sitting or reclining for more than 18 h per weeks (beta: 0.04). It was not significantly correlated with region of habitation (*P* = 0.19) and level of education (*P* = 0.17) (Table 4).

**Table 4:** Multivariable linear regression analysis of the associations between the systolic and diastolic blood pressure and other systemic parameters as dependent parameters in the Ural Eye and Medical Study*.

**Parameter**	**Standardized regression coefficient beta**	**Standardized regression coefficient B**	**95% confidence interval of B**	***P*-value**	**Variance inflation factor**
**SYSTOLIC BLOOD PRESSURE**					
Age: Per unit (years) increase in age	0.27	0.51	0.46, 0.56	<0.001	1.18
Gender (men vs. women)	0.06	2.45	1.35, 3.55	<0.001	1.26
Level of education: Per unit (levels from 0 to 6) increase in educational level	−0.03	−0.46	−0.83, −0.09	0.02	1.14
Blood concentration of triglycerides: Per unit (mmol/L) increase in triglycerides concentration	0.04	1.13	0.45, 1.80	0.001	1.08
Blood concentration of glucose: Per unit (mmol/L) increase in glucose concentration	0.07	0.89	0.59, 1.19	<0.001	1.06
Alcohol consumption (any alcohol consumption vs. no consumption)	0.03	1.42	0.19, 2.65	0.02	1.11
Body mass index: per unit (kg/m^2^) increase in body mass index	0.24	0.98	0.87, 1.09	<0.001	1.19
Waist-hip circumference ratio: per unit increase in waist-hip ratio	0.07	14.4	8.41, 20.4	<0.001	1.26
Sitting or reclining for more than 18 h per week (sitting for >18 h per week vs. sitting for ≤18 h)	0.04	0.001	0.000, 0.001	0.003	1.04
**DIASTOLIC BLOOD PRESSURE**					
Age: Per unit (years) increase in age	0.05	0.05	0.03, 0.08	<0.001	1.18
Gender (men vs. women)	0.11	2.29	1.70, 2.88	<0.001	1.26
Level of education: Per unit (levels from 0 to 6) increase in educational level	−0.03	−0.21	−0.41, −0.02	0.03	1.14
Blood concentration of triglycerides: Per unit (mmol/L) increase in triglycerides concentration	0.05	0.73	0.37, 1.09	<0.001	1.08
Blood concentration of glucose: Per unit (mmol/L) increase in glucose concentration	0.06	0.35	0.19, 0.52	<0.001	1.06
Alcohol consumption (any alcohol consumption vs. no consumption)	0.07	1.66	1.00, 2.32	<0.001	1.11
Body mass index: Per unit (kg/m^2^) increase in body mass index	0.22	0.46	0.40, 0.52	<0.001	1.19
Waist-hip circumference ratio: Per unit increase in waist-hip ratio	0.08	8.55	5.34, 11.8	<0.001	1.26
Sitting or reclining for more than 18 h per week (sitting for >18 h per week vs. sitting for ≤18 h)	0.04	0.000	0.000, 0.001	0.002	1.04

**Figure 3:**
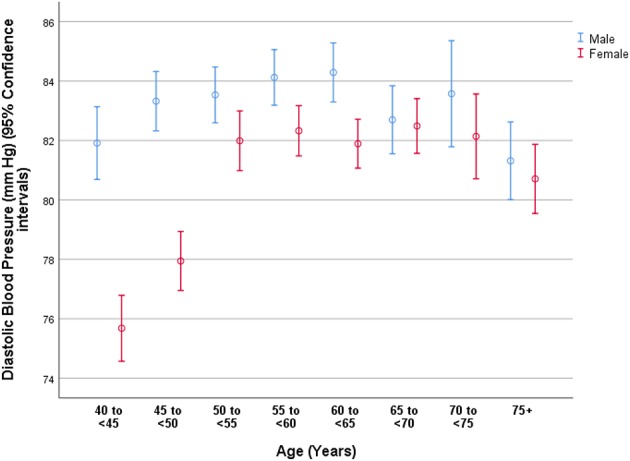
Graph showing the distribution of diastolic blood pressure in the Ural Eye and Medical Study, stratified by age and gender.

Higher DBP was associated (multivariable linear regression analysis) with older age (beta: 0.05) (Figure 3), male gender (beta: 0.11; *P* < 0.001), lower level of education (beta: −0.03), higher blood concentrations of triglycerides (beta: 0.05) and glucose (beta: 0.06), higher prevalence of alcohol consumption (beta: 0.07), higher body mass index (beta: 0.22), higher waist-hip circumference ratio (beta: 0.08), and sitting or reclining for more than 18 h per weeks (beta: 0.04) (Table 4).

## Discussion

The results of our study suggest that in this Russian study population with an age of 40+ years the prevalence of elevated BP/hypertension was 87.3% (95% CI: 86.4, 88.1). This rate was considerably higher than the prevalence of hypertension (53.2%; 95% CI: 51.9, 54.5) when its former definition with a SBP ≥ 140 mmHg and/or a DBP ≥ 90 mmHg was used. The awareness of elevated BP among individuals with elevated BP/hypertension or a history of it was 47.6% (95% CI: 46.0, 49.0), and the rate of therapy among the individuals with elevated BP/hypertension was 21.2% (95% CI: 20.1, 22.3). Among those with anti-hypertensive therapy, only 33 out of 1,022 (3.1%) individuals had normal BP values, while 59 (5.6%) participants had a hypertensive crisis. A higher prevalence of elevated BP/hypertension was associated with older age (*P* < 0.001), male gender (*P* < 0.001), urban region of habitation (*P* = 0.01), lower level of education (*P* = 0.003), higher body mass index (*P* < 0.001), and higher waist-hip circumference ratio (*P* = 0.003), higher prevalence of sitting or reclining for more than 18 h per week (*P* = 0.003), higher prevalence of alcohol consumption (*P* < 0.001), and higher serum concentrations of triglycerides (*P* = 0.01) and glucose (*P* < 0.001).

The relatively high rate of elevated BP/hypertension of 87.3% in this Russian population agrees with the finding obtained in the GBD 2016, that high SBP was the leading mortality risk factor in Russia, causing 32.7% of all deaths (4). Since the present study is one of the first investigations examining the prevalence of hypertension in Russia, the data on the frequency of elevated BP and hypertension obtained in this study cannot directly be compared with the results of other studies on Russian populations. The findings obtained in the present study agree with recent observations made in other countries that, as expected, the prevalence of elevated BP/hypertension was considerably higher if the new definition was applied as compared to the use of the old definition (13, 14). For the National Health and Nutrition Examination Survey, Muntner et al. reported that using the new definition of hypertension increased the crude prevalence of hypertension among US adults (aged 20 years or older) from 31.9% (95% CI: 30.1–33.7%) to 45.6% (95% CI: 43.6, 47.6%).

The prevalence of hypertension in this Russian population [using the old definition: 53.2%; (95% CI: 51.9, 54.5)] was higher than in populations from India, China or Western countries (2, 3, 15–21). Correspondingly, the GBD 2015 reported that Russia, besides China, India, Indonesia, and the United States, belonged to the five countries worldwide which accounted for more than half of all global DALYs associated with a SBP of at least 110–115 mm Hg, although Russia's population is considerably smaller than the population of other countries (2).

Among the individuals with stage 1 or higher of hypertension, the awareness rate was 47.6%, and among the participants with elevated BP or stage 1+ of hypertension, the awareness rate was 45.4%. These figures were lower than those for the majority of high-income countries, and they were similar to those for Ireland (NCD Risk Factor Collaboration). The treatment rate in our study population was 21.2% within the group of participants with stage 1 or higher of hypertension, and it was 20.2% among the individuals with elevated BP or stage 1+ of hypertension. These figures were lower than in any high-income country in the list of which Ireland had the lowest values (men: 39%; men: 50%) (NCD Risk Factor Collaboration). Among the participants of our study with anti-hypertensive therapy, only 3.1% of them had normal BP values. This figure indicated a poor therapeutic control of arterial hypertension (NCD Risk Factor Collaboration). The low rate of successful therapy of hypertension in our study population corresponds to an outpatient health facilities-based survey by Mozheyko et al. who found a BP control rate (<140/90 mm Hg) of 16.8% in a group of 1,794 patients visiting government-run outpatient health facilities in the Yaroslavl Region of Russia, about 280 km north east of Moscow in 2011 (5, 6). Interestingly, the prevalence of severe uncontrolled hypertension (SBP ≥ 180 mm Hg) was 9.7%, while in our study population a hypertensive crisis (SBP > 180 mm Hg and/or DBP > 120) was detected in 2.7% (95% CI: 2.3, 3.1) of the participants.

As in other study population, the prevalence of hypertension was associated with the parameters of older age, male gender, urban region of habitation, lower level of education, higher body mass index and higher waist-hip circumference ratio, higher prevalence of alcohol consumption, a more sedentary life style, and higher serum concentrations of triglycerides and glucose (Table 3). The association between a higher prevalence of arterial hypertension and older age was not linear and men as compared to women had a higher prevalence of hypertension (Figures 1–3). It fits with the observations made in previous studies on other ethnic groups (NCD Risk Factor Collaboration). In particular the DBP showed an increase from the age group of 40 to <45 years to the age group of 65 to <70 years before it declined toward the oldest age groups (Figure 3). The steepness of the increase for the younger age groups was more marked for women than for men, presumably since the women started at a lower level (Figure 3). The decline in the mean DBP within the oldest age groups may be due to various factors, including a higher prevalence of an aortic valve insufficiency in older age and/or a higher mortality of individuals with a higher DBP. A longer survival of individuals with lower DBP would lead to a reduction in the mean DBP in the older age groups.

When we discuss the results of our study, its limitations should be taken into account. First, the study was performed when the previous guidelines for diagnosis and therapy of arterial hypertension were still valid, so that the fraction of arterial hypertensive individuals under therapy will have to be updated in few years when the new guidelines have been generally implemented. Second, the study was based on a single examination instead of a series of arterial blood pressure measurements performed at various daytimes. Third, as for any population-based study, the participation rate is critical to assure the representativeness of the study population. With more than 80% of the eligible population taking part in our investigation, a pronounced bias in the inclusion of participants might have been unlikely. Fourth, our study population was composed of different ethnicities. While this multi-ethnic composition was typical for Southern Russia, the population of North-Western Russia and Central Russia usually shows a higher percentage of Russians. To overcome this potential limitation of our study, we assessed the prevalence of arterial hypertension in dependence of the ethnic background and found that the prevalence did not differ significantly between the Russian group and the non-Russian group. The age and gender distribution in our study population was comparable to the results of the Russian census 2010 (http://www.gks.ru/).

In conclusion, using the new definition provided by the American College of Cardiology/American Heart Association, the prevalence of stage 1 or higher of hypertension in this typically mixed Russian population aged 40+ years was high (87.3%), with an awareness rate of 45.4%, a treatment rate of 20.2, and a low rate of therapeutic control of BP elevation in those individuals under treatment.

## Data Availability Statement

The datasets generated for this study are available on request to the corresponding author.

## Ethics Statement

The studies involving human participants were reviewed and approved by In agreement with the Declaration of Helsinki, the Ethics Committee of the Academic Council of the Ufa Eye Research Institute approved the study protocol and all participants gave informed written consent. The Ethics Committee confirmed that the methods were carried out in accordance with the relevant guidelines and regulations. The patients/participants provided their written informed consent to participate in this study.

## Author Contributions

MB, GK, and JJ: design and conception. MB, GK, RZ, VS, TG, DY, YU, IA, SP-J, SM, RK, SA, IN, AZ, and JJ: data assessment, editing, and final approval of the manuscript. JJ and SP-J: writing of the manuscript.

### Conflict of Interest

The authors declare that the research was conducted in the absence of any commercial or financial relationships that could be construed as a potential conflict of interest.
